# *Leptospira* in Brazilian Bats (Mammalia: Chiroptera): A Systematic Review

**DOI:** 10.1002/vms3.70619

**Published:** 2025-09-29

**Authors:** Carolina dos Santos Braga, Caio Graco Zeppelini

**Affiliations:** ^1^ Escola de Medicina Veterinária e Zootecnia Universidade Federal da Bahia Salvador Brazil; ^2^ Department of Wildlife, Fish and Environmental Studies Swedish University of Agricultural Sciences (SLU) Umeå Sweden

**Keywords:** leptospirosis, neglected tropical disease, One Health, zoonosis

## Abstract

Bats are a megadiverse clade with plural ecological functions, including their role as bacterial, viral and eukaryotic zoonotic pathogen reservoirs. This includes *Leptospira*, a bacterial genus that causes severe aetiologies in humans and animals. However, knowledge on the Bat–*Leptopsira* relationships and diversity lacks systematization, and its coverage is uncertain. This study reviewed the publications related to *Leptospira* infection in bats in the Brazilian territory. Eight studies published between 1976 and 2024 were recovered, conducted in 5 of the 27 federative units of the country. The studies tested a total of 1167 animals of 62 species plus 3 identifications to the level of genus, and one study citing ‘bats’, with 133 individuals of 28 species being positive (plus ‘bats’). Detection was performed using molecular methods (PCR and sequencing), serological tests (microagglutination) or a combination of both. The level of detail and quality of reporting varied significantly between studies. Our results indicate that studies on bat‐borne *Leptospira* are yet to adequately cover bat fauna in Brazil both in terms of diversity and geographic distribution. More studies, with standardized methods for reporting the findings, combining serological and molecular methods, are necessary for a proper characterization of the epidemiological role of bats in Brazil.

**Summary:**

- Eight studies on bat‐borne *Leptospira* were retrieved for Brazil
- Studies cover about one‐third of the known bat diversity for the country
- Studies present low geographic coverage and are skewed towards a small group of species

## Graphical abstract

We performed a systematic review on bats and *Leptospira* in Brazil. 8 studies were conducted between 1976 and 2024, with 66 bat taxa found positive, 1 *Leptospira* species and four serovars identified. Graphical abstract made with Biorender.

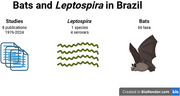

## 1 Introduction

Leptospirosis is a zoonosis caused by bacteria of the genus *Leptospira*. Originally a disease associated with rural settings, leptospirosis is becoming more important in urban settings of the Global South with the disorganized growth of cities and the emergence of slums (Bradley and Lockaby 2023). Current estimates indicate that leptospirosis is responsible for 58,900 human deaths and 1.03 million cases worldwide (Costa et al. 2015). Nonetheless, leptospirosis remains a neglected infectious disease associated with poverty and environmental vulnerability (Costa et al. 2015; Torgerson et al. 2015).

Bacterial infection occurs through contact with water and soil contaminated with the urine of infected animals (Guerra 2009), causing both asymptomatic/mild infections and severe syndromes with high risk of death (Ko et al. 2009). Brown rats (*Rattus norvegicus*) are the main reservoir in urban settings (Bradley and Lockaby 2023; Minter et al. 2018), although several mammal species are susceptible to the infection and can act as reservoir or spreading hosts. In dogs, infections can develop into severe aetiologies such as liver failure, acute kidney damage and hepatitis (André‐Fontaine and Triger 2018). Cats can act as asymptomatic reservoirs and shed bacteria through urine, although vulnerable to developing the disease (Murillo et al. 2020). Serovars associated with cattle and pigs are known to infect humans, with ranch and abattoir workers representing vulnerable populations (Brown et al. 2011). Equines are maintenance hosts (Alves et al. 2016; Arent et al. 2016; Pinna et al. 2011), with reports of uveitis, miscarriages and other clinical manifestations (Divers et al. 2019), although not considered a common infection (Tirosh‐Levy et al. 2021). Given the low specificity of early clinical symptoms/signals in humans and animals, leptospiral infections are considered neglected diseases with complex epidemiology due to close association with wild and domestic species (Browne et al. 2022; Costa et al. 2015; Narkkul et al. 2021).

Bats (Order Chiroptera) are the second most diverse order of mammals, with 1466 species organized in nine families occurring in all continents except for Antarctica, and represent the sole modern non‐avian vertebrate lineage with self‐propelled flight (Zubaid et al. 2006). In Brazil, 186 species occur throughout all ecosystems (Garbino et al. 2024), including urban environments, where 86 species are known to occur (Nunes et al. 2017). The taxonomic diversity of the group is reflected in their variety of ecological roles that include invertebrate population control, pollination and seed dispersal (Kunz et al. 2011), but also include their significant role as pathogen reservoirs (Brierley et al. 2016; Calderon et al. 2016; Castelo‐Branco et al. 2023; Dhivahar et al. 2023; Luis et al. 2013).

Although focus on bat‐borne pathogens is focused on viruses (Calderon et al. 2016; Forero‐Munoz et al. 2024; Pinheiro et al. 2024; Roffler et al. 2024), with particular focus on their role on rabies epidemiology (Belotto et al. 2005; Caraballo et al. 2024; Escobar et al. 2015), bats are important reservoirs of bacterial and eukaryotic pathogens (Castelo‐Branco et al. 2023; de Souza et al. 2023; Ferreira et al. 2024; Franca et al. 2024; Karunarathna et al. 2023). This includes *Leptospira*, of which they are considered asymptomatic carriers, capable of shedding the bacteria through their urine (Brito et al. 2023; Esteves et al. 2022; Ferreira et al. 2024). However, their role on the epidemiology of this bacterial infection remains poorly understood and represents a high‐risk gap in knowledge, especially given the close association fostered by synurbanization (Nunes et al. 2017).

Given the trends of habitat encroachment and increased inter‐species contacts (including with humans), the potential for bat‐related zoonotic pathogen emergence and transmission is a significant One Health concern (Eby et al. 2023; Hayman et al. 2013). To better understand this epidemiological scenario, inform research and policy programs and protect human and animal health, clear and systematic information is key. To address this situation, the present study synthetizes the current information on bat‐borne *Leptosira* in Brazil.

## 2 Materials and Methods

We performed a systematic review on bat‐associated *Leptospira* studies in the territory of Brazil. The scientific publication indexers Scielo (scielo.br), Scopus (scopus.com) and PubMed (pubmed.ncbi.nlm.nih.gov) were selected, using the keyword strings (chiroptera OR “bat” OR “bats”) AND (leptospir*), with the addition of indexer‐specific operators when needed (e.g., the use of TITLE‐ABS‐KEYWORD for Scopus). Queries were performed on 17 October 2024, with results exported to EndNote 20 (Clarivate Analytics) for triage.

Duplicates were removed in two steps: one tool‐assisted step using EndNote's ‘find duplicates’ tool and a manual step, to find duplicates that could not be detected by the program due to differences in formatting between indexers. First triage was performed by analysing the title and abstract of the manuscripts, using as elimination criteria (i) non‐scientific/grey literature and review papers; (ii) studies outside of Brazil; (iii) studies that did not include bats; (iv) studies that did not test for *Leptospira*. The second round of triage was performed by reading the full text of the retained entries, applying the same criteria. Data extraction was performed using Microsoft Excel spreadsheets. All triage steps were performed independently and posteriorly compared.

A supplementary screening was performed in Scielo, using the Portuguese keywords (Morcego* OR quiróptero*) to include manuscripts published in Brazilian Portuguese, with a single result included (Lins and Rosa 1976).

All procedures for this systematic review observed the PRISMA recommendations for best practice in systematic reviews (Page et al. 2021)

## 3 Results

### 3.1 Data Triage

**FIGURE 1:**
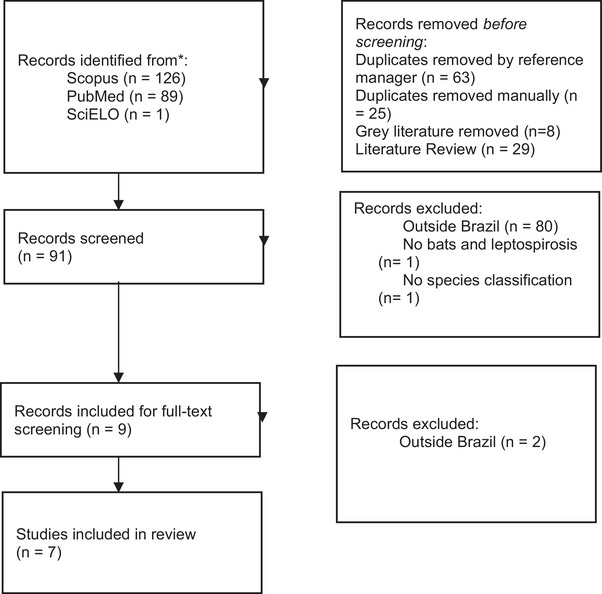
Systematic review diagram.

A total of 91 unique results were recovered from the indexers. A total of 81 studies were removed during triage, resulting in nine articles that were read in full. Seven articles were included in the final data extraction (Figure 1), to which the single result in Brazilian Portuguese was added, resulting in a total of eight studies included in the data extraction.

### 3.2 Temporal and Spatial Distribution of Studies

**FIGURE 2:**
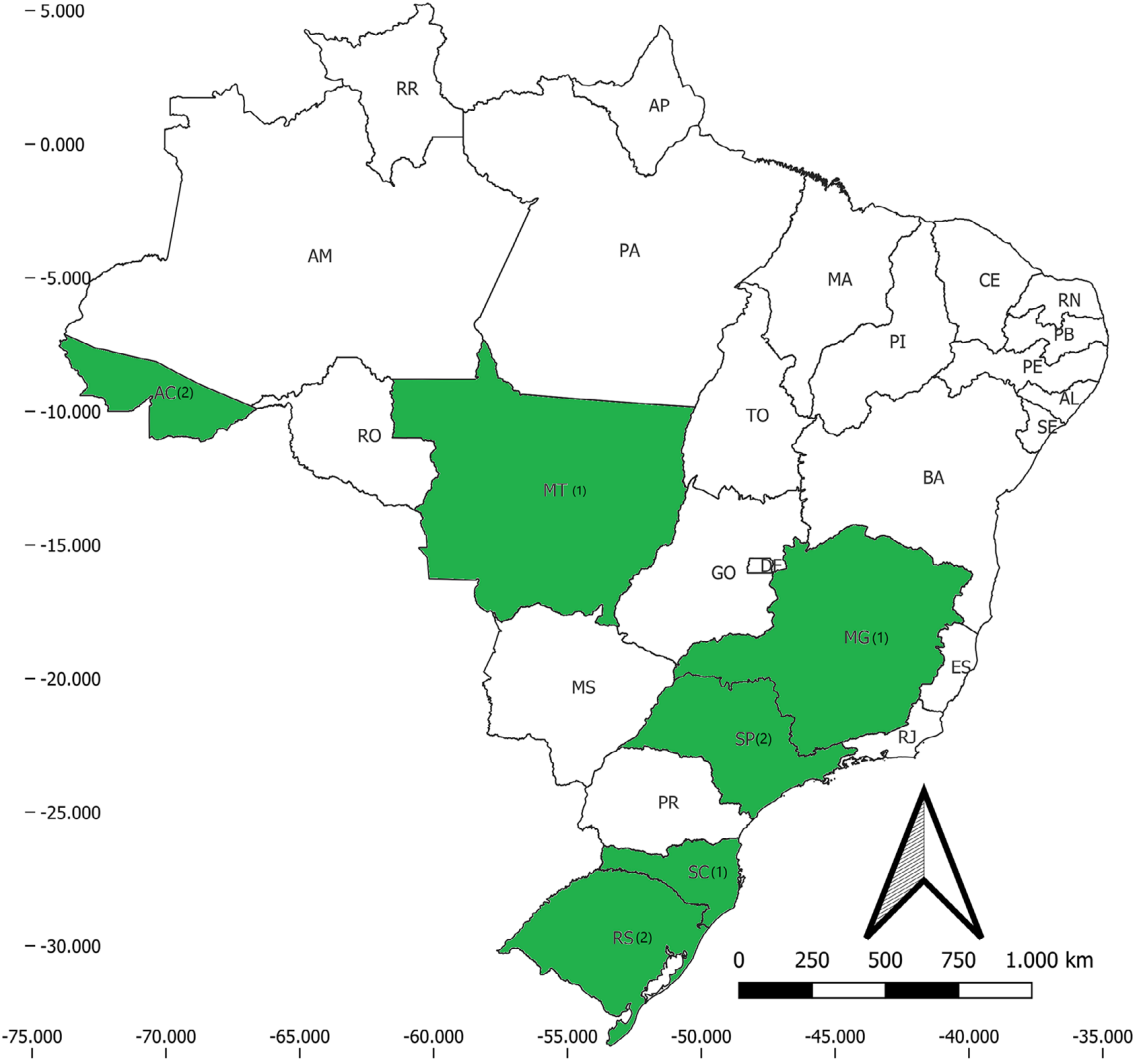
Spatial distribution of the studies included in the review. Values in parentheses indicate the number of studies for each federative unit.

A single study was performed before the year 2000, with most (*N* = 6) being published from 2010 onwards (Table 1). Studies were concentrated in four states of the Southeast and South of Brazil: Minas Gerais (MG), São Paulo (SP), Rio Grande do Sul (RS) and Santa Catarina (SC). No studies were performed in the Northeast, one was performed in the Midwest (MT), and two studies were performed in the state of Acre, Northern Brazil (Figure 2). Three studies were conducted in urban areas, one sampled rural/periurban area, one focused on forest remnants (in urban matrix and a conservation unit), one study covered urban, rural and forest areas, while one study did not specify the environments sampled (Table 1).

**TABLE 1:** Studies included in the data extraction, temporal distribution, regions sampled, diagnostic methods and samples used.

Author/Year	Type of sampled environment	Tissue sample	Diagnostic method
Lins and Rosa (1976)	Not specified	Serum	MAT
Zetun et al. (2009)	Rural	Serum	MAT
Bessa et al. (2010)	Urban	Kidney, serum	PCR and MAT
Mayer et al. (2017)	Urban (convenience sample)	Kidney	PCR
Ferreira et al. (2021)	Urban/periurban	Spleen, liver, blood	PCR and DNA sequencing
Ulsenheimer et al. (2022)	Urban (convenience sample)	Kidney	PCR
Di Azevedo et al. (2023)	Urban forests, conservation units (forest)	Kidney	PCR and DNA sequencing
Verde et al. (2024)	Urban, periurban, rural, forest	Kidney	PCR

### 3.3 Bat Diversity

A total of 1167 bats of 66 taxonomic groups (62 species, 3 identifications to genus level [*Eumops*, *Molossus* and *Myotis*], and two studies citing ‘bats’) were tested. A total of 127 individuals of 29 taxonomic groups (28 species and ‘bats’) were *Leptospira‐*positive (see the Supporting Information). The positives represent 16 species of Phyllostomidae—the most well‐represented family, 7 species of Vespertilionidae and 5 species of Molossidae (15.6% of Brazilian bat species). The most frequent species with positive individuals were *Desmodus rotundus* (18/212, one study does not report the number of tested individuals), *Molossus molossus* (6/149), *Tadarida brasiliensis* (34/124), *Glossophaga soricina* (6/89, one study does not report the number of tested individuals), *Artibeus planirostris* (10/51, two studies do not report the number of tested individuals, with one reporting two positives), *Carollia perspicillata* (5/33, two studies do not report the number of tested individuals, with one reporting one positive) and *Platyrrhinus lineatus* (2/31, one study does not report the number of tested individuals).

One hundred and one individuals of 37 taxonomic groups (35 species and three identifictions to the level of genus) were tested with no positive individuals (see the Supporting Information), representing 24 species of Phyllostomidae, 9 of Molossidae, 3 of Vespertilionidae and 1 of Emballonuridae (see the Supporting Information). Overall, 33.3% of the Brazilian bat diversity was tested for *Leptospira*. One study (Ferreira et al. 2021) had only negative results.

### 3.4 *Leptospira* Detection and Diversity

Six studies applied PCR as a detection method. Three of them with PCR alone, two including DNA sequencing and one also used microagglutination tests (MATs) (Table 1). MAT was used in three studies, two of which applied the test alone. PCRs were performed using kidney (*N* = 5), and one study used spleen, liver and blood samples. DNA sequencing used kidney (*N* = 1), liver, spleen and blood (*N* = 1) tissues. PCR tests targeted the *Leptospira* genes *lipL32* (*N* = 5) (Table 3), *16S rRNA* (*N* = 3) and *secY* (*N* = 2). Studies included methods of double amplification followed by sequencing, used GAPDH as a reference gene for DNA quality assessment, and compared the sequences obtained with data from GenBank.

Regarding the diversity of *Leptospira* species and serovar diversity, serological tests detected the serovars Javanica, Shermani, Pyrogenes and Australis (Table 2). Two molecular studies detected *Leptospira interrogans* (Ulsenheimer et al. 2022; Verde et al. 2024), while the remaining reported only pathogenic *Leptospira* spp. Two studies (Di Azevedo et al. 2023; Verde et al. 2024) indicate potential new species of *Leptospira* based on phylogenetic analysis. *L. interrogans* was identified in 13 species of bats.

**TABLE 2:** Species positive for *Leptospira*.

Species	Diagnostic method	Tissue sample (PCR)	Species/serovar identified
Phyllostomidae			
*Artibeus lituratus*	MAT, PCR, DNA sequencing	Kidney, serum, liver	*Leptospira interrogans*
*Artibeus obscurus*	MAT, PCR, DNA sequencing	Kidney, serum, liver	*Leptospira* spp.
*Artibeus planirostris*	MAT, PCR, DNA sequencing	Kidney, serum, liver	*Leptospira* spp., *L. interrogans*
*Carollia brevicauda*	MAT, PCR, DNA sequencing	Kidney, serum, liver	*Leptospira interrogans*
*Carollia perspicillata*	MAT, PCR, DNA sequencing	Kidney, serum, liver	*Leptospira* spp.
*Choeroniscus minor*	MAT, PCR, DNA sequencing	Kidney, serum, liver	*Leptospira* spp.
*Desmodus rotundus*	MAT, PCR, DNA sequencing	Kidney, serum, liver	*Leptospira* spp., *L. interrogans*, Javanica (200–800), Pyrogenes (100–1600), Shermani (200–800)
*Gardnerycteris crenulatum*	MAT, PCR, DNA sequencing	Kidney, serum, liver	*Leptospira* spp.
*Glossophaga soricina*	MAT, PCR, DNA sequencing	Kidney, serum, liver	*Leptospira* spp., *L. interrogans*
*Lophostoma brasiliense*	MAT, PCR, DNA sequencing	Kidney, serum, liver	*Leptospira* spp.
*Lophostoma silvicolum*	MAT, PCR, DNA sequencing	Kidney, serum, liver	*Leptospira* spp.
*Platyrrhinus lineatus*	MAT, PCR, DNA sequencing	kidney, serum	*Leptospira* spp.
*Rhinophylla fischerae*	MAT, PCR, DNA sequencing	Kidney, serum, liver	*Leptospira* spp.
*Sturnira tildae*	MAT, PCR, DNA sequencing	Kidney, serum, liver	*L. interrogans*
*Tonatia maresi*	MAT, PCR, DNA sequencing	Kidney, serum, liver	*Leptospira* spp.
*Uroderma bilobatum*	MAT, PCR, DNA sequencing	Kidney, serum, liver	*Leptospira* spp., *L. interrogans*
Molossidae			
*Eumops auripendulus*	PCR	Kidney	*Leptospira* spp.
*Molossus currentium*	PCR	Kidney	*Leptospira interrogans*
*Molossus molossus*	PCR	Kidney	*Leptospira* spp., *L. interrogans*
*Molossus rufus*	PCR	Kidney	*Leptospira* spp.
*Tadarida brasiliensis*	PCR	Kidney	*Leptospira* spp, *L. interrogans*
Vespertilionidae			
*Eptesicus diminutus*	PCR	Kidney	*Leptospira* spp.
*Histiotus velatus*	PCR	Kidney	*Leptospira* spp., *L. interrogans*
*Lasiurus blossevillii*	PCR	Kidney	*Leptospira interrogans*
*Lasiurus ega*	PCR	Kidney	*Leptospira* spp.
*Myotis riparius*	MAT, PCR, DNA sequencing	Kidney, serum, liver	*Leptospira* spp.
*Myotis levis*	PCR	Kidney	*Leptospira interrogans*
*Myotis nigricans*	PCR	Kidney	*Leptospira* spp.
Bats^a^	MAT	?	Javanica, Australis

*Note*: Values in parentheses represent the range of titres reported in Zetun et al. (2009).

^a^ Species not reported by the authors.

**TABLE 3:** Genes targeted for molecular diagnostics of *Leptospira*.

Author/year	Target gene
Bessa et al. (2010)	16S rRNA
Mayer et al. (2017)	lipL32
Ulsenheimer et al. (2022)	lipL32
Ferreira et al. (2021)	16S rRNA, lipL32
Verde et al. (2024)	lipL32, secY
Di Azevedo et al. (2023)	16S rRNA, lipL32, secY

### 3.5 Quality Assessment of Reports

All studies present information on the methods used to obtain the samples and the origin of the animals tested, although the level of detail varied considerably. Only one study (Zetun et al. 2009) presents detailed descriptions of the sampling areas. Two studies used convenience samples obtained from rabies testing services (Instituto de Pesquisa Veterinária Desidério Finamor [Mayer et al. 2017] and Centro Estadual de Vigilância em Saúde de Porto Alegre [RS] [Ulsenheimer et al. 2022]), indicating that the animals come from ‘urban centres’, with no further detail. No information on the origin of the samples was provided by Lins and Rosa (1976). Although details on the environments sampled are present, Verde et al. (2024) do not provide information to link individuals captured and environments of origin. One study (Lins and Rosa 1976) does not discriminate the species tested (presented as ‘bats’), with a second one also presenting unidentified samples. Only species with positive results are present in Di Azevedo et al. (2023). One study worked only with *D. rotundus* (Zetun et al. 2009). Verde et al. (2024) presents inconsistencies in the table with the results of the captures and tests (e.g., *Micronycteris microtis* presenting 0 captures, but 1 negative test).

## 4 Discussion

Eight studies from 6 out of the 27 Brazilian federative units were recovered, concentrated in the Southeast–South regions of the country. This region concentrates the majority of studies on bat fauna overall, given the concentration of research institutes and groups (Bernard et al. 2011). The studies were able to cover about a third (62) of the bat species occurring in the country, distributed in 6 families, with circa 15% (28 species from 3 families) being positive. Most species found in the studies are members of the family Phyllostomidae, which is expected given the sampling method used in the studies that sourced individuals from the wild (ground‐level mist‐nets), known for biasing samples towards low‐flying members of the family (Bernard 2001, Bernard et al. 2001). Six serovars were identified by serological methods, while one species (*L. interrogans*) and two potential new species were detected by molecular methods.

The scarcity of studies highlights the neglected status of bat‐related *Leptospira* in Brazil, which could be expected given the underdiagnosed, sub‐notified and neglected status of this infection (Bradley and Lockaby 2023). Despite Brazil being one of the countries with the most studies on animal *Leptospira*, both domestic and wild, in the Americas (Browne et al. 2022), the knowledge gap regarding bats is concerning given their epidemiological relevance (Castelo‐Branco et al. 2023; De Oliveira and Bonvicino 2020; Luis et al. 2013; Moratelli and Calisher 2015) and their high diversity in the country (Garbino et al. 2024), including urban environments (Nunes et al. 2017). These informational deficiencies, however, are expected given the biases on information regarding the clade in Brazil (Aguiar et al. 2020; Bernard et al. 2011), and the tendency of studies on *Leptospira* to focus on synanthropic rodents (Davignon et al. 2023; Vieira et al. 2018).

Overall, based on the studies that report the total of individuals tested, 970 animals were tested, with 127 (circa 13.1% of tested animals) being positive. Other countries in the neotropics presented higher prevalences such as Mexico (21.9%, PCR) (Suarez‐Galaz et al. 2024), (47.3%, PCR, only *D. rotundus*) (Chong‐Guzmán et al. 2025), and Colombia (51.8%, PCR) (Silva‐Ramos et al. 2025), but higher than in Peru (3.4%, PCR) (Matthias et al. 2005). This positivity rate is lower than the mean positive percentage observed in a recent meta‐analysis (26.9%) (Esteves et al. 2022), and could possibly be attributed to the differences in sample sources (freshly‐captured animals, samples from tissue banks), differences in target design (single‐species studies vs. community‐wide studies) and temporality/seasonality. Another source of variation on positivity could be the target tissues as, for example, one study (Ferreira et al. 2021) applied PCR in spleen and liver tissues, with negative results. While other tissues are indicated as potential substrates for molecular detection of leptospires (e.g., urine, saliva and blood [Esteves et al. 2022]), the use of spleen and liver for this finality is uncertain. Pooling results from different detection techniques (PCR for different genes, MATs), representing different epidemiological endpoints (active presence of bacteria vs. antibodies, which might persist after the pathogen is eliminated from the organism), also affect the overall positivity and the comparability between studies. The low number of reports and the high variation in report quality have precluded the application of meta‐analysis techniques. Together with studies that do not properly report the number of individuals tested, the overall positivity we present here should be interpreted carefully in light of the *caveats* above.

The data reported by the study authors varied significantly, with studies lacking details on the sourcing of tested individuals (method of capture, locality details, sampling effort, temporality), taxonomic information of the species (Lins and Rosa 1976) or absence of information on negative species (Di Azevedo et al. 2023), obscuring important information on ecological factors and on the participation of different bat species in the transmission cycle. The use of samples obtained from sample banks, although an important tool for research and increasing territorial coverage of epidemiological studies (Mayer et al. 2017; Ulsenheimer et al. 2022), highlights issues related to the lack of systematic records and metadata associated with the samples, such as date of capture, precise locality and other associated information. Studies applying MATs did not report cut‐off titres in the methods, and only one study (Zetun et al. 2009) provided the titres detected. Only two studies applying molecular tests report sensitivity thresholds (Mayer et al. 2017; Ulsenheimer et al. 2022). These inconsistencies in reporting hinder the capacity of comparison between different studies and the capacity to inform epidemiological analyses and practice, as the contribution of each bat species to the cycle is poorly characterized. Standardized methods on reporting scientific findings, such as the ARRIVE statement (Percie du Sert et al. 2020), could significantly benefit the study of bat‐related *Leptospira*, help deal with the intrinsic variations of studies (such as sampling design and trapping success) and increase the comparability and power of evidence of published manuscripts.

Differences in the reporting of the resolution of the *Leptospira* species and prevalences detected were expected, given that MAT tests allow for the identification of the serovar alone. Molecular tests such as PCR allow for identification of species alone, although taxonomic accuracy depends on several factors such as the quality of the amplification and the availability of sequences for comparison. While, for taxonomic characterization, the combination of serological and molecular methods should be applied, these methods alone are not sufficient to provide robust evidence on the role of bats as effective reservoirs of *Leptospira*, given that they do not provide information on the viability and infectivity of the bacteria potentially being shed (Esteves et al. 2022; Matthias et al. 2005), with proof of viability tests (such as isolation and culture) still necessary.

Our findings point to some interesting tendencies on the epidemiology of bat‐borne *Leptospira*. (i) The remarkable presence of positive bats in urban areas indicates the epidemiological risk of bat‐to‐human transmission and, consequently, the importance of investigation and monitoring, particularly through targeted surveillance studies in areas with endemic transmission or high rates of human cases. Given that bat‐mediated leptospirosis is expected to occur when humans get in contact with substrates contaminated with bat urine, roosts (which often contain guano beds due to bats urinating/defecating), foraging areas (such as orchards, places with concentrations of potential prey animals for sanguinivore bats) represent potential epidemiological risk, and should be prioritized for future investigation. (ii) The use of samples from other epidemiological monitoring services highlights the necessity to capitalize on the sampling effort already performed by the surveillance teams and the availability of tissue samples. It is important to foster the adoption of more robust metadata archiving by these services, which could be achieved by providing them with technical support and training. (iii) There is an overrepresentation/sampling bias towards *D. rotundus*, indicating a negative perception of the species as a risky taxon, potentially due to its sanguinivore habit and perceived epidemiological importance. The habits of *D. rotundus* might also indicate a possible mechanism linking bat fauna and the environmental compartment (water and soil) of the transmission cycle of *Leptospira*. *D. rotundus* walks in the ground during feeding bouts as part of how they approach their prey (Morais and Novaes 2025), potentially getting in contact with soil contaminated with the urine of infected animals, including (potentially) its prey (Zetun et al. 2009). This might represent a route of contact between *Leptospira* and bats, and might transmit to other individuals and/or species in roost or other contact interactions.

While there are some self‐evident recommendations (such as increasing the geographic range of sampling and number of studies) based on the potentialities and limitations observed in the studies that compose this systematic review, a few specific recommendations for future studies can be proposed to improve strength, comparability and epidemiological informativeness:

- Detailed metadata on the origin of tested animals, with characterization of sampled environments, method of capture and sample effort employed and temporality.
- Detailed information on detection methods, with sensitivity or cut‐off thresholds, positive/negative controls. For serological methods, present the panel of serovars used. Whenever possible, combine molecular and serological methods for better taxonomic resolution.
- Present the whole sample, with clear indication on the captured species, the number of samples tested per species, positivity rates and the level of positivity for serological tests, including species with negative results. In case of multiple environments/localities tested, segregate species/samples per source. For studies using previously collected individuals or banks, indicate the temporality of tested samples.
- Make use of other methods for bat sampling (canopy mist‐nets, roost sampling) to increase the taxonomic coverage of studies. Passive sampling methods, such as analysing guano beds, might also provide important insights without needing to capture individuals, with the caveat of its limitations to link to the species of origin and inability to determine the prevalence of infection.

## 5 Conclusions

Bat‐borne *Leptospira* is still a neglected research subject in Brazil, lacking adequate geographic and taxonomic coverage, and varying quality in the information reported. Given the polysemic importance of bats for infectious disease epidemiology, particularly in urban settings, more studies on bat–*Leptospira* relationships are urgently needed, particularly expanding the taxonomic diversity and geographic coverage, with the adoption of robust standardized methods for sampling, testing and reporting their findings.

## Author Contributions

**Carolina dos Santos Braga**: data curation, investigation, writing. **Caio Graco Zeppelini**: conceptualization, data curation, investigation, writing, supervision.

## Conflicts of Interest

The authors declare no conflicts of interest.

## Peer Review

The peer review history for this article is available at https://www.webofscience.com/api/gateway/wos/peer‐review/10.1002/vms3.70619.

## Supporting information




**Supplementary material 1**: – Data on bats captured, tested and their outcomes, as extracted from the original manuscripts. In cases of divergence between the text of the study and the tables, the tables were prioritized for this summary.? = the number of individuals is missing. X = not all individuals captured were tested, so it is not possible to infer the proper number of tested individuals per species.

## Data Availability

No data were created or used for this study.
